# Treatment for Patients With Malignant Pheochromocytomas and Paragangliomas: A Perspective From the Hallmarks of Cancer

**DOI:** 10.3389/fendo.2018.00277

**Published:** 2018-05-28

**Authors:** Camilo Jimenez

**Affiliations:** Department of Endocrine Neoplasia and Hormonal Disorders, The University of Texas MD Anderson Cancer Center, Houston, TX, United States

**Keywords:** pheochromocytoma, paraganglioma, tyrosine kinase inhibitors, radionuclides, immunotherapy

## Abstract

Malignant pheochromocytomas and paragangliomas affect a very small percentage of the general population. A substantial number of these patients have a hereditary predisposition for the disease and consequently, bear the risk of developing these tumors throughout their entire lives. It is, however, unclear why some patients with no hereditary predisposition develop these tumors, which frequently share a similar molecular phenotype with their hereditary counterparts. Both hereditary and sporadic tumors usually appear at an early age, and affected people often die before reaching their expected lifespans. Unfortunately, there is currently no systemic therapy approved for patients with this orphan disease. Therefore, pheochromocytomas and paragangliomas are very challenging malignancies. The recognition of genetic and molecular abnormalities responsible for the development of these tumors as well as the identification of effective therapies for other malignancies that share a similar pathogenesis is leading to the development of exciting clinical trials. Tyrosine kinase inhibitors, radiopharmaceutical agents, and immunotherapy are currently under evaluation in prospective clinical trials. A phase 2 clinical trial of the highly specific metaiodobenzylguanidine, iobenguane ^131^I, has provided impressive results; this radiopharmaceutical agent may become the first approved systemic therapy for patients with malignant pheochromocytoma and paraganglioma by the United States Food and Drug Administration. Nevertheless, systemic therapies are still not able to cure the disease. This review will discuss the development of systemic therapeutic approaches using the hallmarks of cancer as a framework. This approach will help the reader to understand where research efforts currently stand and what the future for this difficult field may be.

## Introduction

Pheochromocytomas and paragangliomas are neuroendocrine tumors derived from the paraganglia. Most pheochromocytomas and sympathetic paragangliomas secrete excessive amounts of catecholamines that predispose to elevated blood pressure, palpitations, sweats, anxiety, and gastrointestinal disease (1, 2). Patients are prone to develop a catecholamine crisis characterized by a hypertensive emergency and cardiovascular events. The excessive secretion of catecholamines is confirmed by measuring the plasma concentrations of metanephrines (3). Most patients have localized tumors and subsequently, they are cured with surgery (4, 5).

Malignant pheochromocytomas and paragangliomas are rare endocrine cancers. Approximately 100–200 new cases are diagnosed every year in the United States (6). The definition of these malignancies rests on the presence of metastases because there is currently no histological, biochemical, molecular, or genetic marker that can clearly differentiate benign from malignant tumors (7, 8). Therefore, the World Health Organization has recommended classifying pheochromocytomas and paragangliomas as metastatic or nonmetastatic, as a substantial number of patients with clinical predictors of metastases can be diagnosed and treated before the malignant cells spread to distant sites (7, 9). Metastatic pheochromocytomas and paragangliomas (MPPGs) frequently spread to regional and distant lymph nodes, bones, liver, and lungs (10, 11). Metastases are rarely found in the pancreas, breast, central nervous system, or skin (12). As expected, patients with MPPG have shorter overall survival (OS) durations than do patients with non-MPPGs (10).

Patients with MPPG are mainly treated with systemic chemotherapy and radiopharmaceutical agents such as conventional ^131^Iodine-metaiodobenzylguanidine (13). The understanding on these treatments is difficult as it mainly derives from small, retrospective studies (14). Subsequently, there are no guidelines for the treatment of patients with MPPG. Progress toward systemic therapies for patients with MPPG has been slow (13) owing to the rarity of the disease and the lack of a reliable animal model that can mimic a human MPPG phenotype (15, 16). However, the recognition of the fundamental genetic and metabolic characteristics of MPPG and clinical experience with other cancers that share similar pathogenetic processes have led to the identification of new therapeutic horizons (16–18). Approximately 30% of patients with MPPG harbor a germline mutation of the succinate dehydrogenase subunit B of the mitochondrial enzymatic complex 2 gene (*SDHB*) (19). Tumors with *SDHB* mutations are characterized by abnormal angiogenesis and a hypervascular phenotype (20). *SDHB* tumors also display intense DNA hypermethylation and upregulation of the epithelial-to-mesenchymal transition, which fosters distant spread (21–23). In addition, these tumors express cell membrane glucose transporters and activate glucose phosphorylation to support their energetic demands (24). Because *SDHB*-associated MPPGs are very avid for glucose, positron emission tomography with fludeoxyglucose (FDG-PET) is a sensitive test to identify the disease (25). Interestingly, many patients with MPPG do not harbor germline mutations; their tumors are considered apparently sporadic. However, many apparently sporadic tumors exhibit a very similar molecular phenotype to the one observed in *SDHB*-associated tumors (20). In addition, gastrointestinal stromal tumors and renal cell clear cell, medullary thyroid, and pancreatic neuroendocrine carcinomas share some crucial pathogenetic characteristics with MPPG (26–29). Therapeutic progress on these tumors has helped in identifying potential therapies for patients with MPPG (30).

Scientific efforts have identified several biological capabilities, called the “hallmarks of cancer,” that are essential for the formation of cancer in humans (31). These hallmarks are distinctive and complementary abilities acquired by cancer cells that enable tumor growth and metastatic dissemination. Cancer cells have the ability to sustain proliferative signaling, evade growth-inhibiting signals, evade apoptosis, enable replicative immortality, sustain angiogenesis, and invade and metastasize (31). In addition, they can reprogram energy metabolism and evade immune destruction (31). They provide a solid conceptual foundation for understanding the biology of cancer (31). As in other cancers, the survival of MPPG cells likely depends on a combination of these hallmarks. This review will discuss the development of systemic therapeutic approaches for patients with MPPG using the hallmarks of cancer as a framework. We will also assess the value of surgical resection and traditional therapies such as chemotherapy for patients with MPPG.

## Surgery

The early resection of a pheochromocytoma or a sympathetic paraganglioma may cure the disease. In fact, more than 90% of patients with nonmetastatic disease treated with surgery are alive 5 years after initial diagnosis (10). The surgical approach (i.e., open laparotomy or laparoscopy) must be carefully selected on the basis of the presence of clinical predictors of aggressiveness, such as the size and location of the primary tumor and the presence of *SDHB* mutations (32). In patients with subdiaphragmatic primary tumors larger than 5 cm, an open laparotomy allows better visualization of the lymph nodes and is associated with a lower risk of tumor rupture than are laparoscopic procedures (32).

Over the last 20 years, clinical experience has suggested that it may be best to observe most patients with head and neck paragangliomas (33). Because of their parasympathetic origin, it is exceedingly rare to find a head and neck paraganglioma that secretes noradrenaline; consequently, these patients are not prone to hormonal syndromes. In addition, these tumors are rarely metastatic (34) and subsequently, no TNM staging has been proposed yet for head and neck paragangliomas (30). Most importantly, their intimate contact with neurovascular structures increases the risk of intraoperative vascular accidents and postoperative low cranial nerve neuropathy (35).

Patients with MPPG will most likely not be cured by surgery unless they present with only regional lymph node metastases or small, localized, and resectable distant metastases. Nevertheless, patients with noncurable MPPG may still benefit from surgical resection of the primary tumor (32). Resection of the primary tumor may decrease the catecholamine surge associated with these tumors and improve hormonal symptoms (32); patients may consequently have a lower risk for cardiovascular and gastrointestinal morbidity. Furthermore, resection of the primary tumor is associated with an improvement in OS regardless of performance status, tumor burden, genetic profile, or hormonal status (32), likely because of a lower rate of metastatic spread, as patients exhibit similar OS rates irrespective of their hormonal status (32).

## Chemotherapy

Understanding the role of chemotherapy in patients with MPPG is challenging. Chemotherapy decreases the tumor’s ability to sustain proliferative signaling, which underlies its abnormal cell growth and division. However, chemotherapy does not induce complete responses; in fact, retrospective studies have shown variable responses. The difficulties faced by clinicians are highlighted by a recent systematic review and meta-analysis of all published studies on the topic of chemotherapy for MPPG (14). Of 459 potential studies, only 4 (<1%) were of high enough quality for inclusion in the meta-analysis (36–39). These four studies included consecutive patients, had an adequate description of diagnostic and therapeutic interventions, employed a clear definition of and evaluation criteria for tumor response, and had few or no lost patients during follow-up. The results of this meta-analysis suggested that approximately 37% of patients with MPPG respond to systemic chemotherapy with a combination of cyclophosphamide, vincristine, and dacarbazine (14). Patients generally did not have complete responses. However, some had improved blood pressure control and apparent improvement in the symptoms of catecholamine excess attributable to a reduction in tumor size or stabilization of disease (14). Only one study—the largest one—suggested that MPPG patients whose tumors responded to chemotherapy had longer OS than did patients without a tumor response (36). This study was also the only one that clearly indicated chemotherapy for patients with progressive disease (36). Therefore, the results of this meta-analysis may have overestimated the rate and scale of MPPG response to chemotherapy (14). Toxicity related to chemotherapy varies and duration of therapy has not been determined yet. A maintenance regimen with dacarbazine or temozolomide may improve chemotherapy long-term efficacy (16, 40).

## The Hallmarks of Cancer and MPPG

The tumor growth observed in patients with MPPG clearly demonstrates that sustained proliferative signaling allows the excessive activation of the cell division cycle in these tumors. As in other malignancies, this process is in part mediated by tyrosine kinase receptors. The interaction of growth factors with these receptors activates signaling pathways that modulate the cell cycle and cell growth; these signals also control cell survival and energy metabolism (20). MPPGs are frequently characterized by a tumor environment of pseudohypoxia, which leads to deregulation of cellular energetics, abnormal activation of proliferative pathways, tumor inflammation and necrosis, and activation and recruitment of cells that prevent immune system recognition of the tumor. MPPGs associated with *SDHB* mutations and other MPPGs associated with an environment of pseudohypoxia (i.e., those with germline mutations in regulatory genes of the other subunits of the mitochondrial enzymatic complex 2, fumarase, or the protein von Hippel-Lindau disease) exhibit a phenotype characterized by large intratumor concentrations of vascular endothelial growth factors (VEGFs), platelet-derived growth factor beta (PDGF-β), epidermal growth factors, fibroblast growth factors, and others; their cognate receptors are also overexpressed by these tumors (20, 41). The stabilization of the hypoxia-inducible factor (HIF) under conditions of pseudohypoxia is responsible for the overexpression of genes responsible for the synthesis of these growth factors and their receptors (20). Hereditary mutations also confer advantages to specific clones that benefit from, for instance, the activation of epigenetic mechanisms such as DNA hypermethylation (22, 42). Mutations of the *EPAS1* gene, which codes for the HIF-2α, have been described in 6% of patients with pheochromocytoma and paraganglioma and strongly suggest a pathogenic and, therapeutically speaking, targetable role for hypoxia (43, 44). This gene controls several proteins involved in cell division, angiogenesis, and red blood cells production (43). In addition, somatic activating mutations of certain receptors may result in structural modifications that lead to independent signaling activation. Recently, activating mutations of the c-Met receptor have been described in MPPG (45).

Along with the sustained proliferative signaling supported by pseudohypoxia, MPPG cells exhibit deregulation of cellular energetics (46). These tumors compensate for pseudohypoxia with increased expression and activity of glucose transporters and glycolytic regulatory enzymes such as hexoquinases and pyruvate and lactate dehydrogenases (17). The resulting negative energetic balance may lead to necrosis, which in turn leads to activation of inflammatory cells that contribute to tumor cell growth and angiogenesis. Neoangiogenesis, necrosis, and inflammation, DNA hypermethylation, and other mechanisms activate the epithelial-to-mesenchymal and mesenchymal-to-epithelial transition pathways that lead to the development of metastases (31). Activating c-Met mutations may also facilitate the distant spread observed in some MPPGs (45).

Somatic mutations in other genes may also activate downstream pathways. For example, *RAS* mutations have been described in pheochromocytomas and paragangliomas (47, 48). These mutations predict a constitutive activation of the mitogen-activated protein kinase and phosphoinositide 3-kinase (PI3K) pathways, leading to abnormal proliferation of pheochromocytoma and paraganglioma cells. *RAS* mutations have not, however, yet been associated with a clear MPPG phenotype (47, 48).

Metastatic pheochromocytomas and paraganglioma cells require unlimited replicative capacity in order to create a macroscopic tumor. In fact, MPPGs are frequently characterized by large primary tumors and massive metastases (32). This implies that MPPGs are able to overcome the two mechanisms that prevent cell immortality: senescence and death/crisis. The telomeres that protect the ends of the chromosomes are strongly involved in the regulation of this hallmark (31). The maintenance of telomeric DNA is linked with tumor cell immortalization. The polymerase telomerase extends telomeric DNA by adding telomere repeat segments, preventing senescence; the loss or erosion of telomeres may trigger the senescence process (49). Recently, somatic mutations of telomerase have been identified in MPPG (50). In addition, noncanonical roles of telomerase and its subunit telomerase reverse transcriptase may also contribute to the development of MPPG (50). Mutations in *ATRX* which is involved in chromatin remodeling have been described in some MPPG tumors (51, 52).

In order to survive, MPPG cells also require mechanisms that allow them to evade apoptosis and immune system recognition. TP53-inactivating mutations have been described in some MPPGs, and abnormal activation of the PI3K and mechanistic target of rapamycin pathways has also been observed in MPPGs (18, 53). Somatic *NF1* mutations may inhibit autophagy in MPPG cells (54). Recently, a great deal of interest in oncology has been focused on the identification of therapies that may enhance the immune system recognition of tumor cells. Several mechanisms that prevent immune system recognition have been described in cancers characterized by hypoxia and pseudohypoxia (55).

**Figure 1:**
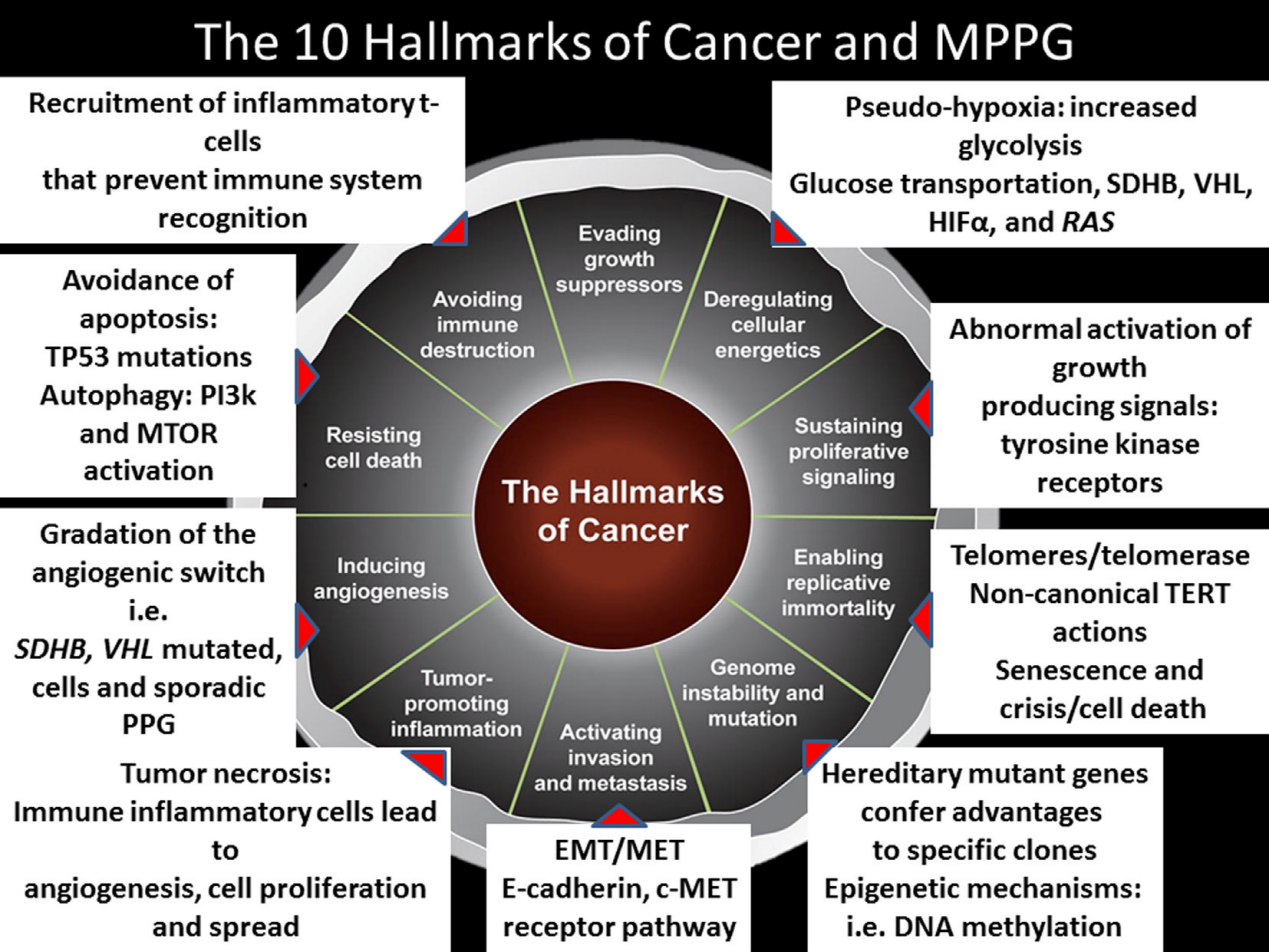
The 10 hallmarks of cancer and metastatic pheochromocytomas and paragangliomas (MPPGs). This figure describes the 10 hallmarks of cancer and several mechanisms identified to date in patients with MPPG that contribute to their tumor development.

It is important to emphasize that like in other malignancies, the microenvironment determines the joint success of the hallmarks of cancer (56). Preclinical studies have shown that the production of lactate by cancer activated fibroblasts stimulates the migration of *SDHB* silent pheochromocytoma cells (57). Furthermore, clinical evidence reveals that MPPG cells—irrespective of their genotype—are very much attracted by the bone microenvironment (58). As it will described later, this finding supports exploring medications such as cabozantinib for patients with bone metastases. Figure 1 summarizes the hallmarks of cancer in the context of MPPG.

## Novel Therapies for MPPG According to Their Effects on One or More Hallmark Capabilities

### Inhibition of Angiogenesis and Proliferative Signaling: Pazopanib and Sunitinib

Pazopanib and sunitinib block the VEGF-1, -2, and -3, PDGF-α and -β, c-Kit, fms-related tyrosine kinase 3, and ret proto-oncogene (RET) receptors. As such, these medications prevent neoangiogenesis, cell growth, and cell migration and may induce apoptosis (59, 60).

Sunitinib was the first tyrosine kinase inhibitor recognized as a potential treatment for patients with MPPG (61). A retrospective study of 17 patients treated with sunitinib provided useful information that has helped in designing prospective trials (62). These patients did not have a response to chemotherapy or had contraindications to chemotherapy. Thirteen patients had measurable disease, and four patients had predominant bone metastases. Three patients discontinued sunitinib therapy early because of adverse events and were not evaluable for objective response; 10 patients with measurable disease were evaluable, and the objective response rate (ORR) was 30%. In addition, one patient had stable disease with some degree of regression. Patients with partial responses and stable disease also saw improvement in their symptoms of catecholamine excess; their blood pressure normalized, and two patients discontinued all antihypertensive therapies for some time. Six patients had no response to sunitinib. The four patients with nonmeasurable disease (bone metastases) exhibited a substantial reduction of glucose uptake as assessed *via* FDG-PET imaging and had improved blood pressure control (62).

Sunitinib treatment had clinical benefits in both carriers of *SDHB* mutations and patients with apparently sporadic tumors (62). The progression-free survival (PFS) (the length of response duration) was, however, not very impressive (4.1 months). The study was in fact an intention-to-treat analysis that included the three nonevaluable patients in the final evaluation of PFS. Some patients, nonetheless, exhibited a durable response to sunitinib; the longest response lasted 3 years. The study concluded that some patients with MPPG may benefit from antiangiogenic therapies such as sunitinib (62). However, the dose of sunitinib must be carefully chosen, and adverse events should be prevented or treated aggressively with dose adjustment and/or interventions to prevent exacerbation of hypertension or symptoms such as pain. The FIRSTMAPPP trial (NCT01371201) is a multinational phase 2 study evaluating sunitinib in patients with MPPG. The intervention group receives 37.5 mg sunitinib daily. This dose of sunitinib is lower than the dose currently approved for the treatment of patients with kidney cancer (50 mg daily, 4 weeks on, 2 weeks off). The lower dose may be associated with a better safety profile.

Pazopanib was tested in a phase 2 clinical trial involving patients with MPPG (63). The intervention group received 400 mg pazopanib daily for 2 weeks of the first cycle, then 800 mg for 2 weeks of the second cycle, followed by 800 mg daily for the whole of the subsequent cycles. Patients needed to have measurable disease, as the primary endpoint was ORR. Of the seven recruited patients, only one patient exhibited a confirmed partial response of −57%. This patient’s response to pazopanib lasted for approximately 2 years. Four patients had disease progression. Almost every patient exhibited hypertension; severe hypertension was noted in 50% of patients, including one patient who developed Takotsubo cardiomyopathy. The serious cardiovascular adverse events happened once the dose of pazopanib was titrated to 800 mg daily. The trial was terminated because of poor accrual (63).

This trial aimed to evaluate pazopanib as a potential therapeutic option for MPPG patients because previous comparative studies in patients with kidney cancer suggested that pazopanib was better tolerated than sunitinib (64, 65). Later, however, it was recognized that these studies had several pitfalls related to patient selection and quality-of-life assessment (66); more importantly, clinical practice indicated that patients treated with sunitinib and pazopanib had, in reality, similar compliance patterns. Thus, clinical considerations, including the physician’s experience, should determine treatment choices. Pazopanib could prove to be an effective medication to treat MPPG. Nevertheless, it is important to remember that MPPGs are very challenging tumors; in addition to a large tumor burden, MPPGs frequently secrete excessive amounts of catecholamines that predispose patients to cardiovascular disease. A pazopanib dose of 800 mg daily was likely too high to tolerate.

### Inhibition of Angiogenesis: Axitinib

Pseudohypoxia activates angiogenesis through stimulating the synthesis and secretion of VEGFs by MPPG cells. Axitinib is a potent antiangiogenic medication that has been approved by regulatory agencies for the treatment of patients with kidney cancer. Axitinib inhibits VEGFR-2 but does not inhibit other receptors involved in angiogenesis such as PDGFR-β. For this reason, axitinib is expected to cause fewer adverse events than multi-tyrosine kinase inhibitors that also target angiogenesis (67).

A phase 2 clinical trial of axitinib (NCT01967576) enrolled 11 patients (68). The primary endpoint was PFS, and secondary endpoints included ORR and safety. The intervention was 5 mg axitinib given twice daily. The dose of axitinib was increased to 7–10 mg twice daily in patients who did not experience side effects more severe than grade 2 hypertension. Approximately 36% of patients achieved a partial response, and 54% had stable disease. Of those with stable disease, half had some degree of regression. Only one patient exhibited disease progression. The ORR was 36% (68). No patients tolerated the starting dose of 5 mg twice daily for a long period of time because they developed hypertension. Hypertension was common and frequently serious. About 82% of patients had grade 3–4 hypertension and required dose reduction or discontinuation of therapy (68). The trial is currently closed for recruitment.

### Inhibition of Angiogenesis, Proliferative Signaling, and Invasion and Metastasis: Cabozantinib

Cabozantinib is a multi-tyrosine kinase inhibitor approved for the treatment of patients with medullary thyroid and clear cell renal cell carcinomas. Cabozantinib is perhaps, the most potent antiangiogenic medication available in clinical practice. Cabozantinib inhibits VEGFR-2 as well as the RET and c-Met receptor pathways (69, 70). Although the inhibition of the RET receptor pathway may not be of interest for the treatment of the great majority of patients with MPPG (malignant pheochromocytomas are an exceptional phenotype of patients with multiple endocrine neoplasia type 2, which is associated with RET mutations) (71), the inhibition of the c-Met pathway may indeed be of interest. MET activation is a universal mechanism that drives cell survival, invasion, and metastasis in many cancers and cooperates with the VEGFR pathways to promote tumor angiogenesis (72). Upregulation of the c-MET pathway occurs as a consequence of the VEGFR inhibition, leading to tumor resistance to antiangiogenic medications and escape from VEGFR inhibition (72). Inhibition of the c-Met pathway may delay the development of tumor resistance and improve clinical outcomes (73). Patients with kidney cancer treated with cabozantinib exhibit significantly longer PFS than do patients treated with sunitinib (74). Cabozantinib is also an interesting medication to study in MPPG patients because of its potential impact on the bone microenvironment. Cabozantinib has been associated with palliation of bone pain, improvement of anemia, modulation of bone turnover, and bone scan resolution in patients with malignancies frequently associated with bone metastases (75). MPPG frequently spreads to the bones, predisposing patients to overwhelming skeletal-related events (58, 76).

A phase 2 clinical trial of cabozantinib is currently ongoing (NCT02302833). The primary endpoint of this study is ORR. The trial includes an exploratory branch of patients with MPPG with predominant bone metastases. The intervention is 60 mg cabozantinib daily with dose titration to 40 or 20 mg depending on patients’ toleration of adverse events. Patients require objective evidence of disease progression to be included in the trial. Preliminary results in 10 patients with measurable disease showed an ORR of 40% (77). Half of patients had stable disease, and only one patient did not have a response to therapy. All patients with stable disease had tumor regression; the clinical benefit rate was 90% (77). In addition, all patients with bone metastases exhibited a reduction of glucose uptake as demonstrated by FDG-PET. Patients with hormonally active tumors associated with partial responses or stable disease exhibited improvement of symptoms of catecholamine excess, including diabetes mellitus and hypertension (77). No patients experienced severe hypertension. However, most patients required dose reduction because of grade 2 fatigue or hand-foot syndrome. Two patients required dose reduction because of asymptomatic grade 3 elevation of pancreatic enzymes and formation of a rectal fistula, respectively (77). This clinical trial is actively recruiting participants.

### Induction of Cell Death and Prevention of Replicative Immortality: Iobenguane ^131^I and ^177^Lu-DOTATATE

The radiopharmaceutical metaiodobenzylguanidine (MIBG) was created in 1979 (78). MIBG is labeled with ^131^I at the meta-position and is taken up by the noradrenaline transporter. Once inside the tumor cell, MIBG releases lethal radiation that causes severe DNA damage, inhibiting cell proliferation and causing cell death. Up to 80% of MPPG patients have tumors that express the noradrenaline transporter in the cell membrane (79). Responses to MIBG are, however, limited, with only 30% of MPPG patients seeing a clinical benefit (80). The limited benefits associated with MIBG are likely attributable in part to its manufacturing process (81). MIBG is produced by a simple isotope exchange that leaves a large amount of unlabeled MIBG, called cold MIBG, in each dose. Cold MIBG may compete with labeled MIBG for the noradrenaline transporter, preventing the uptake of labeled MIBG and decreasing the tumor’s exposure to radiation. MIBG delivers low levels of radioactivity per dose (~1.59 MBq/μg) (82). In addition, cold MIBG may compete with noradrenaline for the noradrenaline transporter, increasing the concentrations of circulating noradrenaline and predisposing to cardiovascular events during or shortly after the drug’s administration (83). Iobenguane ^131^I is also MIBG labeled with ^131^I at the meta-position. Unlike conventional MIBG, iobenguane ^131^I is produced from a solid-phase ultratrace precursor that eliminates the presence of cold MIBG. Iobenguane ^131^I is, then, a highly specific radiopharmaceutical agent that delivers very high levels of radioactivity per dose (~92.5 MBq/μg). Furthermore, iobenguane ^131^I may be associated with a lower rate of cardiovascular events than conventional MIBG (84).

A phase 1 dose-escalation study of iobenguane ^131^I in patients with MPPG determined the maximum tolerated dose to be 296 MBq/kg (8 mCi/kg) (85). A pivotal phase 2b clinical trial of iobenguane ^131^I was then developed, with an intervention of 2–500 mCi doses of iobenguane ^131^I separated by a period of at least 3 months depending on bone marrow toxicity. This trial recruited 81 patients, 68 of whom had MIBG-avid tumors and received at least one therapeutic dose of iobenguane ^131^I. Fifty patients received two doses of iobenguane ^131^I (86). The trial’s primary endpoint was clinical: the number of patients who had at least a 50% reduction in the dose and number of antihypertensives for at least 6 months. Secondary endpoints included ORR, OS, and safety (86). One-quarter of patients achieved the primary endpoint, and many of the patients who did not achieve the primary endpoint nonetheless had improvement of hypertension with a reduction of less than 50% in the dose and number of antihypertensives (86). Almost all patients had a tumor response. Partial responses and stable disease were noted in 30 and 68%, respectively, of patients treated with two doses (86). The proportion of patients who experienced a partial response increased over time, suggesting that iobenguane ^131^I has persistent antitumor effects (86). Overall, 90% of patients treated with two doses continued to have a partial response or stable disease 12 months after receiving the initial dose (86). The most common treatment-emergent adverse events were consistent with expected radiation-related risks: bone marrow suppression, nausea and vomiting, fatigue, and dizziness. Hematological toxicities resolved within 4–8 weeks and without the need for stem cell transplantation (86). On the basis of these findings, the United States Food and Drug Administration granted breakthrough therapy and fast-track designation to iobenguane ^131^I for the treatment of MPPG.

Peptide receptor radionuclide therapy (PRRT) is a molecular therapy used to treat neuroendocrine tumors. Examples of PRRT agents include ^177^Lu-DOTATATE and ^90^Y-DOTATE. ^177^Lu-DOTATATE is approved for the treatment of patients with somatostatin receptor-positive gastroenteropancreatic neuroendocrine tumors. This radiopharmaceutical binds to the somatostatin receptors present at the tumor cell membrane, delivering lethal radiation. MPPGs usually express somatostatin receptors. In fact, the sensitivity of ^68^Ga-DOTATATE positron emission tomography/computed tomography imaging in patients with MPPG seems to be higher than that of MIBG scans (87, 88). This observation makes ^177^Lu-DOTATATE an interesting medication to evaluate in clinical trials. Initial prospective studies of ^177^Lu-DOTATATE and ^90^Y-DOTATE included occasional patients with MPPG. Response rates were disappointing, with less than 10% of these patients exhibiting a clinical benefit (89, 90). This observation contrasted with the higher sensitivity of octreotide scintigraphy compared with MIBG scans. Investigators hypothesized that MPPGs may have inappropriate expression of somatostatin receptor subtypes and/or processing errors that caused the lack of response to octreotide and its analogs (91). In fact, molecular studies in a few MPPG specimens have found minimal or no expression of the somatostatin receptor 2 (92). DOTATATE mainly targets this receptor (93). Recently, however, the interest in ^177^Lu-DOTATATE for the treatment of patients with MPPG has been reactivated. A retrospective study of patients with MPPG treated with MIBG (*n* = 16), ^90^Y-DOTATE (*n* = 12), or ^177^Lu-DOTATATE (*n* = 2) suggested that PRRT offered better OS and PFS than did conventional MIBG (94). Nevertheless, this study had several limitations. The sample size was very small, the treatment groups for comparison were quite heterogeneous, and the authors did not conduct a multivariate or propensity score analysis to reduce bias. Nevertheless, individual clinical observations suggested that some patients benefited from PRRT (94). In a more recent retrospective study of 20 patients with MPPG treated with ^177^Lu-DOTATATE, 29% had partial responses and 62% had stable disease 3 months after therapy (95). Fourteen patients had hypertension, and only six patients had disease progression before treatment was provided. Nine patients received radiosensitizing chemotherapy. Some reduction in the dosage of antihypertensive medications was observed in 62% of the patients with hypertension (95). However, the small and heterogeneous sample of this study, the fact that most patients had stable disease before treatment, and the simultaneous use of chemotherapy hinder the interpretation of this study’s results. Therefore, a prospective clinical trial is required to prove the efficacy of PRRT in patients with MPPG.

### Regulation of Cellular Energetics: HIF Inhibitors

Several medications that inhibit the HIF-2α pathway have been tested in patients with cancer. Most of these medications have been shown not to be very effective and have not moved from phase 1 to phase 2 clinical trials (96). Crystallography identified a large protein cavity within the HIF-2α PAS-B domain. This cavity is the target of potent 130 HIF-2α inhibitors (PT2385 and PT2977) (97). Recently, PT2385 was evaluated in a phase 2 clinical trial for heavily pretreated patients with progressive renal cancer (98). Several patients had partial responses, and one patient had a complete response. Interestingly, side effects were minimal (98). These drugs have not been tested in patients with MPPG. An investigator-initiated proposal for a phase 2 clinical trial of PT2977 for MPPGs is under evaluation by regulatory agencies.

### Enhancement of Immune System Tumor Recognition: Interferon and Pembrolizumab

Pseudohypoxia causes inactivation of cytotoxic T-cell lymphocytes, activation of immune-suppressive monocytes, increased adenosine production, and increased expression of the immune checkpoint protein programmed death-ligand 1 (PD-L1) and its receptor, among many other immune system disarrangements (55, 99, 100).

One of the first immunotherapies introduced to clinical practice was interferon alpha-2b. Interferon alpha-2b activates natural killer cells that can recognize and destroy cancer cells and has been used for the treatment of patients with gastroenteropancreatic neuroendocrine tumors, melanoma, and kidney cancer. Some of these patients had clinical benefits, with disease stabilization and occasional partial responses (101). These findings correlated with histological evidence of tumor necrosis. For many years, interferon alpha-2b and octreotide analogs were considered the pillars of treatment for patients with gastroenteropancreatic neuroendocrine tumors (102). As MPPGs are also neuroendocrine tumors, occasionally patients were also treated with interferon alpha-2b. A recent retrospective study of 14 patients with progressive MPPG who were treated with interferon alpha-2b showed that 12 patients had disease stabilization and 2 had partial responses (103). Baseline PFS was 9.4 months, while the PFS of patients treated with interferon alpha-2b was 17.2 months. This study suggested that immunotherapy could have a positive impact on patients with MPPG (103). Prospective studies of interferon alpha-2b in other malignancies had several methodological problems (101); furthermore, the side effects of this drug, including fatigue, depression, flu-like syndrome, renal failure, and liver toxicity, substantially alter patients’ quality of life and frequently lead to therapy discontinuation (104). Consequently, interest in interferon alpha-2b has declined.

Over the last decade, several novel immune therapies have been developed. These medications target immune-related molecular pathways such as the cytotoxic T-lymphocyte-associated protein 4 and PD-L1/PD-1 pathways. These pathways, among others, play an important role in the recognition of the cancer cell by the immune system. These immune checkpoint inhibitors are approved for the treatment of several malignancies, including melanoma, kidney cancer, and non-small cell lung cancer. Prospective studies have demonstrated disease stabilization, partial responses, and sometimes disease resolution (105–107). Although serious adverse events—mainly autoimmune events—have been described, for the most part patients tolerate these medications well (105–107). A phase 2 clinical trial of the PD-1 inhibitor pembrolizumab for patients with MPPG is currently underway. This study hypothesizes that the administration of single-agent pembrolizumab to patients with PD-L1-positive MPPG will result in a nonprogression rate of greater than 20% (based on RECIST 1.1 criteria) at 27 weeks (nine cycles). The study is actively recruiting patients (NCT02721732).

## Where are We Going?

Clinical and basic research studies have revealed that replicative immortality, upregulated and sustained proliferative signaling, genome instability (single mutations), inflammation, deregulation of cellular energetics, angiogenesis, and activation of mechanisms responsible for invasion and metastasis are hallmarks of the development of MPPG that could be effectively targeted with available treatments. MPPG treatments, then, can be categorized on the basis of their actions on one or more of these hallmark capabilities. As detailed above, each potential therapy for patients with MPPG typically targets only one or two hallmark capabilities. Preliminary results from prospective clinical trials and observations derived from retrospective studies strongly suggest that angiogenesis is a predominant druggable hallmark of MPPG. Patients treated with angiogenesis inhibitors have indeed exhibited clinical benefits such as tumor size reduction and improvement of hormonal symptoms. However, although individual clinical responses may sometimes last for several years, these responses are, in general, transitory (108). The development of resistance is not exclusive to therapies that target angiogenesis; it is also expected with other therapies because they target a limited number of MPPG hallmark capabilities (109). In fact, cancer cells may, over time, acquire other capabilities that lead to treatment resistance, tumor recurrence, and disease progression.

In addition, each of the hallmark capabilities is regulated by partially redundant molecular pathways, and individual targeted therapies may not fully downregulate all of the molecular pathways responsible for a specific hallmark capability. Therefore, some MPPG cells may survive and adapt over time to the biological impositions of targeted therapies. These adaptive mechanisms may include the development of new mutations, the remodeling of cells’ epigenetic characteristics, and modifications of their microenvironment. Single medications or combined therapies that target several hallmark capabilities at the same time may produce a higher rate of response and more durable benefits. However, patients receiving such treatments might be more prone to develop severe adverse events than are patients treated with medications that target only one or two capabilities. Therefore, the dose and administration of these drugs must be carefully calibrated in order to achieve the best possible therapeutic response while minimizing the severity of side effects. The phase 2 clinical trial of cabozantinib discussed above, for instance, is exploring this concept. It is also important to develop clinical trials that combine therapies with different and complementary mechanisms of action. There are currently no such trials for patients with MPPG, and any such trial will likely need to be preceded by a phase 1 trial to evaluate dosage and safety.

**Figure 2:**
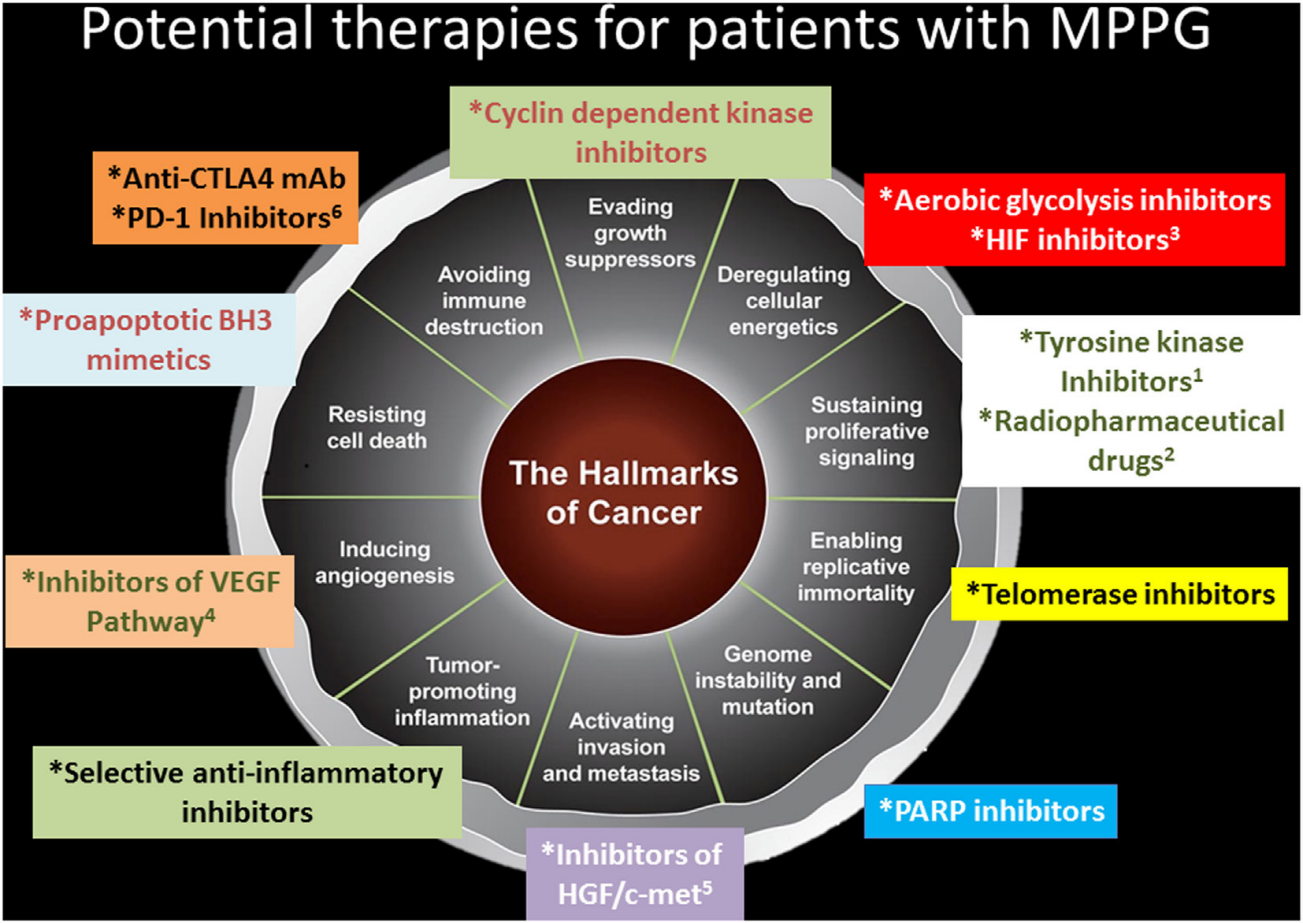
Potential therapies for patients with metastatic pheochromocytomas and paraganglioma (MPPG). This figure describes potential therapies for patients with MPPG. Some of these therapies are currently evaluated in clinical trials: ^1^axitinib, cabozantinib, lenvatinib, pazopanib, and sunitinib; ^2^iobenguane ^131^I, ^177^Lu-DOTATATE; ^3^PT2977; ^4^axitinib, cabozantinib, lenvatinib, pazopanib, and sunitinib; ^5^cabozantinib; and ^6^pembrolizumab.

Our current knowledge about the hallmarks of cancer suggests that exploring other therapeutic options for patients with MPPG may be helpful. Telomerase inhibitors that can modulate replicative immortality, poly (ADP-ribose) inhibitors that can stabilize the genome, selective anti-inflammatory medications, inhibitors of the hepatocyte growth factor and c-Met pathways that can stop invasion and metastasis, proapoptotic Bcl-2 homology domain 3 inhibitors that can prevent resistance to cell death, cyclin-dependent kinase inhibitors that can enhance the activity of growth suppressors, aerobic glycolysis inhibitors, and epidermal growth factor receptor inhibitors that can prevent sustained proliferative signaling all are potential medications to evaluate in clinical trials (Figure 2).

Metastatic pheochromocytomas and paraganglioma is an orphan disease with no Food and Drug Administration-approved therapies. The final results of the phase 2 pivotal clinical trial with iobenguane ^131^I are sound and impressive (86). Given the rarity of MPPG, it would be very difficult to develop a phase 3 clinical trial, so regulatory agencies should consider approving iobenguane ^131^I for the treatment of patients with MIBG-avid MPPG. If such approval is granted, iobenguane ^131^I may become the first-line treatment for many patients with MPPG. Clinical trials with multi-tyrosine kinase inhibitors, HIF inhibitors, ^177^Lu-DOTATATE, and pembrolizumab would become therapeutic options to explore in patients with non-MIBG-avid MPPG, patients with MIBG-avid tumors that do not respond to iobenguane ^131^I, and patients who have contraindications for iobenguane ^131^I therapy.

## Conclusion

The available treatments for MPPG are, so far, not curative; research is needed to evaluate therapies with novel mechanisms of action. The use of tyrosine kinase inhibitors, radionuclide agents, and immune therapy may improve the outcomes of patients with MPPG and should be studied in clinical trials. It is always important to treat and prevent hormonal complications and symptoms that derive from direct drug toxicity, so drug doses must be carefully selected. Clinical trials combining therapies that target several hallmarks of MPPG in a simultaneous or sequential manner are an essential goal of MPPG research.

## Author Contributions

CJ has written this invited review manuscript. CJ created the manuscript structure, abstract, sections, figures, and chose the references. CJ is the only author of this manuscript.

## Conflict of Interest Statement

The author declares that the submitted work was not carried out in the presence of any personal, professional, or financial relationships that could potentially be construed as a conflict of interest.
